# Benefits of Physical Exercise as Approach to Prevention and Reversion of Non-Alcoholic Fatty Liver Disease in Children and Adolescents with Obesity

**DOI:** 10.3390/children9081174

**Published:** 2022-08-05

**Authors:** Valeria Calcaterra, Vittoria Carlotta Magenes, Matteo Vandoni, Clarissa Berardo, Luca Marin, Alice Bianchi, Erika Cordaro, Giustino Simone Silvestro, Dario Silvestri, Vittoria Carnevale Pellino, Cristina Cereda, Gianvincenzo Zuccotti

**Affiliations:** 1 Department of Internal Medicine, University of Pavia, 27100 Pavia, Italy; 2 Pediatric Department, “Vittore Buzzi” Children’s Hospital, 20154 Milan, Italy; 3 Laboratory of Adapted Motor Activity (LAMA), Department of Public Health, Experimental Medicine and Forensic Science, University of Pavia, 27100 Pavia, Italy; 4 Department of Biomedical and Clinical Science, Università degli Studi di Milano, 20157 Milan, Italy; 5 Research Department-LJA-2021, Asomi College of Sciences, 2080 Marsa, Malta; 6 Department of Rehabilitation, Città di Pavia Hospital, 27100 Pavia, Italy; 7 Department of Industrial Engineering, University of Tor Vergata Rome, 00133 Rome, Italy; 8 Neonatal Screening and Metabolic Disorders Unit, V. Buzzi Children’s Hospital, 20154 Milan, Italy

**Keywords:** steatosis, obesity, pediatrics, exercise, physical activity, prevention

## Abstract

Non-alcoholic fatty liver disease (NAFLD) is an important health concern during childhood; indeed, it is the most frequent cause of chronic liver diseases in obese children. No valid pharmacological therapies for children affected by this condition are available, and the recommended treatment is lifestyle modification, usually including nutrition and exercise interventions. In this narrative review, we summarized up-to-date information on the benefits of physical exercise on NAFLD in children and adolescents with obesity. The role of exercise as non-pharmacological treatment was emphasized in order to provide recent advances on this topic for clinicians not deeply involved in the field. Several studies on obese children and adults confirm the positive role of physical activity (PA) in the treatment of NAFLD, but to date, there are no pediatric randomized clinical trials on exercise versus usual care. Among the pathogenic mechanisms involved in the PA effects on NAFLD, the main players seem to be insulin resistance and related inflammation, oxidative stress, and gut dysbiosis, but further evaluations are necessary to deeply understand whether these factors are correlated and how they synergistically act. Thus, a deeper research on this theme is needed, and it would be extremely interesting.

## 1. Introduction

Non-alcoholic fatty liver disease (NAFLD) is an important health concern during childhood; indeed, it is the most frequent cause of chronic liver diseases in obese children [1,2]. Further, it should be reported that having NAFLD in childhood could increase the risk to develop hepatocellular carcinoma in adulthood [3].

NAFLD represents a spectrum of pathological conditions ranging from steatosis to nonalcoholic steato-hepatitis (NASH) and fibrosis [4]. The accumulation of liver fat mainly depends on imbalances between the influx of fatty acids to the liver and the liver’s ability to export these fatty acids in the form of very-low-density lipoprotein (VLDL). Several factors, such as genetic susceptibility, epigenetic mechanisms, gut–liver axis, hormone levels, sedentary lifestyle, and hypercaloric diets [4], have been considered in pathogenesis of NAFLD.

NAFLD diagnosis is not straightforward, as this condition may be asymptomatic until the progression to end-stage liver disease [5]. The diagnosis of the disease was based on any diagnostic method, including liver enzymes, ultrasound, magnetic resonance, and liver biopsy.

Nowadays, no valid pharmacological therapies are available for children affected by this condition, and the recommended treatment is lifestyle modification, usually including nutrition and exercise interventions [3,6,7,8]. Physical activity has been included in the focus of research in children and adolescents with NAFLD either as the only intervention or in association with other lifestyle approaches [6,9].

In this narrative review, we summarized up-to-date information on the benefits of physical exercise on NAFLD in children and adolescents with obesity. The role of exercise as non-pharmacological treatment was emphasized in order to provide recent advances on this topic for clinicians not deeply involved in the field.

## 2. Methods

As a literature narrative review [10], we present a non-systematic summation and analysis of literature available on the topic of benefits of physical exercise as an approach to NAFLD in children and adolescents with obesity. Inclusion criteria to refine the narrative review were: articles in the English language; original scientific papers, clinical trials, meta-analyses, and reviews published on a specific topic in the last 15 years, up to June 2022; and case reports or series and letters were not considered. The authors assessed the abstracts of the available literature (n = 206) and reviewed the full texts of potentially relevant papers (n = 106) that were analyzed and critically discussed. The reference list of all articles was checked to consider relevant studies. The terms adopted for the research, alone and/or combined, are non-alcoholic fatty liver disease, obesity, adolescents, children, physical exercise, childhood obesity, complications, and physical activity. The databases *PubMed, Scopus*, *Web of Science,* and *EMBASE* were used from for research purposes.

## 3. Childhood Obesity and Non-Alcoholic Fatty Liver Disease

### 3.1. Childhood Obesity

Childhood obesity has become one of the major public health concerns not merely in the U.S. and in the Western countries but also in many other areas worldwide [11].

Before the outbreak of the COVID-19 pandemic, obesity prevalence in children and adolescents had plateaued in many wealthy countries, while its levels had risen in low- and middle-income countries [11]. However, during the pandemic, academics have reported a different trend: obesity and overweight in children has started to increase in the richest countries as well.

According to this, the U.S. data show the effects of the pandemic on the obesity trends. As reported by Centers for Disease Control and Prevention (CDC) [12], just before the COVID outbreak, the prevalence of obesity was equal to 19.7% for U.S. children and adolescents in the age group 2–19 years. Furthermore, obesity prevalence was 12.7% for age group 2–5, 20.7% for age group 6–11, and 22.2% for age group 12–19. Instead, only 1 year after the pandemic outbreak, the prevalence of childhood obesity in the U.S. increased to 22.4% in the age group 2–19 years based on a longitudinal cohort of 432,302 persons [13]. The total amount of obese children in the U.S. had a monthly increase of 0.07% a month before the pandemic. This coefficient rose to 0.37% (five times higher) just a month after the virus appeared [13,14].

Risk factors for obesity in the earlier stages of life have a twofold nature. Firstly, it is crucial to take into account the role played by biological risk factors, which include age, gender, and genetic predisposition. Secondly, one must consider the impact of environmental factors in early life, including as dietary intake, physical activity, and sedentary behavior throughout childhood [15].

**Figure 1:**
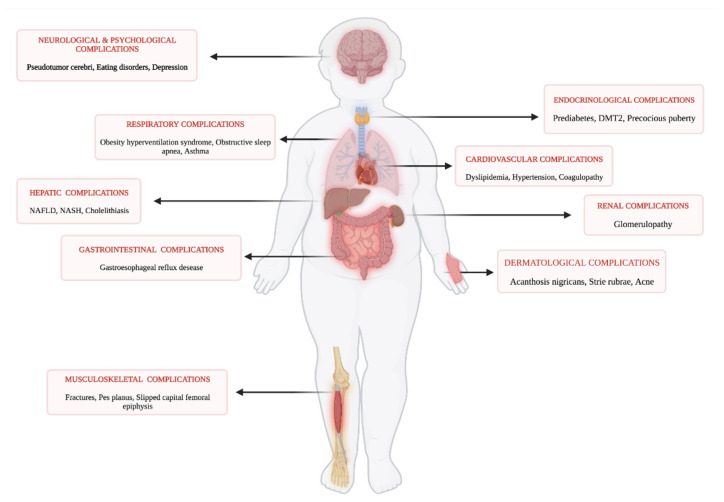
Multiorgan complications related to obesity in children and adolescents (created with BioRender.com, accessed on 1 July 2022).

Childhood obesity is at the root of multiple systemic comorbidities. These include, among others, unbalances in the endo-metabolic system (e.g., type 2 diabetes mellitus, dyslipidemia), respiratory system (e.g., obstructive sleep apnea, asthma), and gastrointestinal system including NAFLD and its evolutions as well as at the cardiovascular and musculoskeletal systems [16,17,18,19,20] (Figure 1). The severity of these comorbidities increases proportionally to the degree of obesity [21].

Prevention remains the best approach to face the burden of this condition. Lifestyle interventions are feasible and effective in both the prevention and treatment of childhood obesity, and pharmacological and surgical treatment are available for a selected population of children and adolescents with obesity [17,18,22,23,24].

A multidisciplinary approach, including physical activity programs, nutrition education, and behavioral modification with highly qualified specialists, represents a key point for the approach to childhood obesity.

### 3.2. Epidemiology of NAFLD in General Pediatric Population and in Children with Obesity

NAFLD is defined as a chronic disease characterized by an accumulation of fat in the liver in the absence of excessive alcohol consumption or other known liver diseases [25], which initially manifests as steatosis in at least 5% of hepatocytes but which can progress to NASH, cirrhosis, and, in severe cases, to hepatocellular carcinoma [26]. It is mainly associated with an incorrect lifestyle and obesity [27] as well as other metabolic disorders such as dyslipidemia and DMT2 [28].

However, the presence of NAFLD can be detected in a minority of lean patients with impaired metabolic status [29]. In these subjects, it seems that genetics have a significant impact [30,31,32]; for example, it has been reported that the s738409 polymorphism of PNPLA3, which results in a loss of the hydrolytic function of the protein and leads to an accumulation of intrahepatic triglycerides and the development of insulin resistance (IR) [33], is higher in non-obese patients than in obese patients [34].

The growing diffusion of obesity in modern society has led to a parallel increase in the incidence of NAFLD, currently statistically widespread in about a quarter of adults worldwide [27]. NAFLD is an increasingly important health problem also among children and adolescents, where it represents one of the most common causes of chronic liver disease [35,36]. Specifically, according to the data collected, the global prevalence is 7.6% in the general pediatric population, with an increase of up to 34.2% in the pediatric population with obesity [37].

An analysis by the National Health and Examination Survey, conducted during the period 2007–2010, found a 6.9% prevalence of NAFLD in a large sample of American adolescents aging from 12 to 19 years old; considering the group of obese adolescents, the prevalence of NAFLD rose from 13% to 27% among obese females, and it rose from 29.5% to 48.3% among obese males from 1988–1994 to 2007–2010 [38].

The Child and Adolescent Trial for Cardiovascular Health (CATCH) reported a prevalence of NAFLD of 36% in Hispanic obese subjects, of 22% in Caucasian obese ones and of 14% in African-American obese ones [39].

Moreover, two Italian studies deployed ultrasound to assess the prevalence of suspected fatty liver among obese children. The results showed echogenic liver in 53% and in 42% of prepubertal and pubertal children, respectively [40].

A study conducted in Malaysia underlined a NAFLD prevalence of 63.3% within obese and overweight children’s groups, with a diagnosis made via ultrasonographic evidence of fatty liver [41]. The 62% of the NAFLD group showed metabolic syndrome based on the definition by the International Diabetes Federation (IDF) [41]. An interesting cross-sectional study was conducted on obese and overweight Iranian children: in these patients, abdominal ultrasound was used to evaluate liver echogenicity. In the research, NAFLD was diagnosed in 8.5% of children with overweight and 24.9% obesity. The NAFLD prevalence was 22.3% for patients aged 6–11 years and 35.5% for patients aged 12–19 years, without significant difference between girls and boys [42].

Differences in prevalence relative to gender have been reported, with a higher incidence rate in males [26] but also with a greater risk of developing fibrosis in females [43,44], as well as regarding age [45] and in relation to the geographical area; in Asia, the prevalence is 10.2% among children [46]; in Europe, it is about 2.5% [47], while in the Hispanic population, it rises up to 11.8% [48]. It seems that Mexican descendants are more predisposed, while young Black individuals seem to have genetic characteristics that protect them from the accumulation of liver fat [27,35,38].

However, it is necessary to underline that the literature lacks data on the pediatric population and that the studies on which those are based are not recent. Furthermore, the inability to make a precise and definitive diagnosis, caused by the limitations imposed by the type of population under consideration, could lead to an underestimation of the real incidence rate of NAFLD among children and adolescents [49]. Finally, the different diagnostic methods used in the studies conducted could influence the prevalence estimate [50].

### 3.3. Pathogenesis and Risk Factors of NAFLD

NAFLD is a complex and multifactorial disease [51,52,53]. At first, the hepatic fat accumulation and the oxidative stress were believed to be the two driving forces causing NAFLD, hence the name of the “two-hit” theory [46]. According to this model, NAFLD is characterized by fat deposition in liver parenchyma, caused when lipid uptake and disposal are not well-balanced: while *de novo* lipogenesis (DNL) is stimulated, β-oxidation and secretion of VLDLs are impaired [53,54,55]. Free fatty acid accumulation causes the liver to be more susceptible to the “second” hit, represented by oxidative stress, mitochondrial dysfunction, stellate cells activation, and pro-inflammatory cytokines imbalance. All these insults can worsen the damage, leading to the progression of non-alcoholic steatohepatitis and fibrosis. However, this “two-hit” hypothesis has been recently replaced by the “multi-hit” theory, which takes into account also the impact of nutritional elements, gut microbiota, and genetic and epigenetic factors [54]. It is well-known that children and adolescents ingest high quantity of sugars, especially fructose, which are responsible for lipid accumulation and DNL. Furthermore, the genetic predisposition could promote NAFLD pathogenesis as well as affect its severity [55]. In fact, genome-wide association studies identified genetic variants associated with NAFLD in several fatty acid genes [56,57,58,59], among which are PNPLA3, KLB, MBOAT7, TM6SF2, GCK, HSD17B13, PPP1R3B, IRGM, and LPIN1 [59,60,61,62]. Fundamental in nature are the genetic variants of the PNPLA3 (also known as adiponutrin) and, in particular, the amino acid substitution p.I148 M of the PNPLA3 gene. Studies show that this variant is associated with hepatic fat content, increased serum liver enzymes, and, moreover, an increased risk of NASH and the progression of fibrosis [59]. Although the biological mechanisms are not exactly defined, the common I148M variant impairs the function of the enzyme with a secondary compromise of lipid catabolism. The association between I148M variant and steatosis was confirmed in obese children in different studies [63,64,65,66].

Besides genetics, epigenetic mechanisms, such as DNA methylation, histone modification, and non-coding RNA regulation, have been gaining more and more interest in NAFLD understanding [56].

The role of gut microbiota (GM) in NAFLD pathogenesis has recently been taken into account. In NAFLD, a GM alteration and enhanced gut permeability determine a major liver exposure to gut-derived bacterial products such as lipopolysaccharides, activating the signaling pathways involved in liver inflammation and fibrogenesis [63,64].

Besides primary NAFLD, associated with insulin resistance, metabolic syndrome, obesity, and DMT2, it is important to also mention secondary causes of NAFLD, including medications, inborn errors of metabolism (IEM), and prenatal factors [65,66]. It has been reported that prolonged administration of aspirin as well as antibiotics can induce pediatric NAFLD [67,68]. In addition, the chronic use of antidepressant and antiepileptic drugs affects body weight and lipid metabolism, stimulating hepatic steatosis both in adults [69] and in children [70,71]. Several IEM, among which are glycogen and lysosomal storage diseases and fatty acid oxidation disorders, if diagnosed late or untreated, can lead to NAFLD [72]. Recent evidences have shown that prenatal factors may predispose to NAFLD. In fact, an association between maternal BMI and newborn hepatic fat accumulation has been established in both animal models [73] and human studies [74]. In addition, preterm birth, intrauterine growth retardation, and low and high birth weight are considered risk factors for severe steatosis in childhood [75,76,77].

### 3.4. Diagnosis of NAFLD

Many of the patients presenting NAFLD are asymptomatic, and therefore, it is difficult to promptly intervene to prevent the disease from progressing. Thus, NAFLD should be suspected in all children who are overweight and in adolescents with a waist circumference > 95th percentile for sex and age [78]. According to what was reported by the ESPGHAN Hepatology Committee, in children over 10 years with predisposing factors, NAFLD is probably attributable to obesity, while for those who are younger, a detailed diagnostic evaluation is required to exclude any other causes of hepatic steatosis [79]. In any case, according to the guidelines published in 2012, the panel of examinations for the differential diagnosis of NAFLD should contain: complete blood count, biochemical profile, coagulation test, glycosylated hemoglobin, glucose tolerance test, thyroid function, serum copper, ceruloplasmin, 24 h copper in urine, liver autoantibodies, A1 antitrypsin levels, viral hepatitis antibodies, sweat test, anti-transglutaminase A and total IgA, serum lactate, amino and organic acids, free fatty acids plasma, acylcarnitine profile, creatine phosphokinase (CPK), and history of drug treatment.

However, as reported in some studies, BMI does not reflect true body composition [80], while aminotransferases appear to have low sensitivity and specificity in NAFLD screening [81]. Indeed, it has been shown that the levels of ALT and aspartate aminotransferase (AST) fluctuate during the disease and do not correlate with fibrosis [82,83], and the serum ALT cutoff has not yet been precisely defined in the pediatric population [84]. Therefore, it is possible to consider the evaluation of these parameters as a support rather than as the main diagnostic reference [44]. Indeed, the presence of hepatic steatosis must be confirmed by ultrasound or by liver biopsy [43,85].

Hypertriglyceridemia is another biochemical marker frequently reported in obese children with NAFLD [86], with a positive correlation with an increased ALT [87,88]. In children’s atherogenic lipid profile, this correlates with the severity of liver injury [89]. Increased serum uric acid level has been also reported in the majority of subjects with the metabolic syndrome and has been proposed as an independent predictor of NAFLD both in adults [90] and children [91].

Liver biopsy represents the gold standard for the diagnosis of NAFLD and allows to determine traces of steatosis, fibrosis, or a local inflammatory state [92]. Furthermore, it allows to exclude the presence of any other liver diseases, such as autoimmune hepatitis [36,79]. However, it turns out to be quite invasive and not without risks [36,79], which are facts that make it a complicated examination to perform in the pediatric population and an unethically adoptable option in the field of research. However, histological analysis of liver biopsies allows to diagnose the extent of steatosis and subsequently to grade the pathology as mild, moderate, and severe.

Furthermore, some studies seem to associate it with a high rate of false negatives [44,93]. Therefore, the first-line test to date is represented by ultrasound [94]; available, non-invasive, and convenient, it has a sensitivity of 85% and a specificity of 95% [44] although, according to the systematic review by Awai HI et al., the positive predictive value of NAFLD varies between 47% and 62% [95].

Among other instrumental tests, computed tomography allows to observe the liver in its entirety and to evaluate moderate or severe states of steatosis. Considered superior to simple ultrasound, it has a sensitivity of 82% and a specificity of 100% [96].

Conventional magnetic resonance imaging (MRI) can also be used to determine minor steatosis states [97] but not to diagnose NASH or to evaluate fibrosis [98]. The magnetic resonance imaging-derived proton density fat fraction (MRI-PDFF) allows to overcome the problem and to identify any degree of steatosis [99] with high sensitivity and specificity, while the magnetic resonance spectroscopy (MRS) can detect steatosis with a percentage greater than 5% with good diagnostic reliability [100]. However, the validity of these techniques is still to be investigated [95,101].

The evaluation of specific markers such as IL-8, monocyte chemoattractant protein-1, resistin, soluble IL-1 receptor I, soluble IL-2 receptor α, and TNF-α [93] as well as serum panels related to liver fibrosis, such as FibroTest and FIB4 index, although the latter have been tested mainly in adults [102,103], has recently been suggested.

Finally, three scoring systems are currently in use to determine the severity of the disease: the Brunt system [104], the NASH CRN system [105], and the SAF system [106].

### 3.5. Treatments of NAFLD

Since NAFLD may progress to cirrhosis and end-stage liver disease [26], it is fundamental to treat patients as soon as possible. NASPGHAN and ESPGHAN guidelines recommend lifestyle modification (see below), which is considered the only effective treatment for NAFLD in both adults and children [107].

A diet low in carbohydrates, fructose, saturated fatty acids, and trans-fatty acids [92,108,109,110] and an active lifestyle are the key components of treatment of NAFLD. BMI reduction is associated to a significant improvement of ALT and histological feature [111,112].

Physical activity as the only intervention or in connection with other lifestyle proposal was recently reported in children with NAFLD, and it will be explained in detail in the next sections.

Unfortunately, pharmacological therapies for pediatric steatosis, such as the administration of metformin, an insulin sensitizer, or the supplementation with the antioxidant vitamin E, are limited and with contrasting results [111,113]. Other options that revealed conflicting effects include the treatment with docosahexaenoic and eicosapentaenoic acids as sources of omega-3 fatty acids, which might inhibit lipogenesis and promote fatty acid oxidation and reduce inflammation, and probiotics aimed to restore gut microbiome [114,115]. Although novel molecules are actually under investigation in adults (obeticholic acid, an analog of bile acids; elafibranor, an insulin sensitizer; liraglutide), no ongoing pediatric trials evaluates the efficacy of these drugs in children [113].

In selected morbidly obese adolescents in which bariatric surgery could be indicated after growth completion, and only as a part of multidisciplinary treatment, reported studies show that the weight loss after bariatric surgery results in NASH and fibrosis reversal [26,116,117].

**Figure 2:**
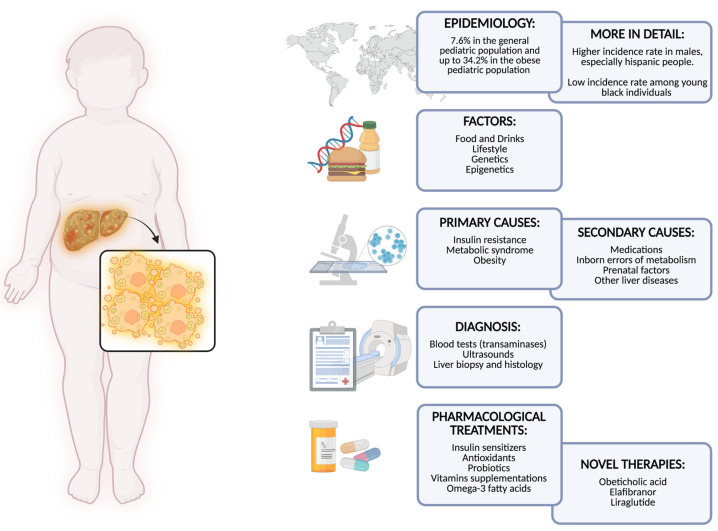
Nonalcoholic fatty liver disease in children and adolescents (created with BioRender.com, accessed on 1 July 2022).

In Figure 2, epidemiology, pathogenicity and risk factors, and diagnostic and therapeutical approaches are explained.

## 4. Physical Activity Levels in Children and Adolescents with Obesity

Regular PA practice is crucial for correct development and growth of children [118,119], and it has a main role in the prevention of overweight and obesity during childhood and adolescence, decreasing the health risks related to weight gain [16]. The current guidelines by the World Health Organization (WHO) recommend to perform at least 60 min of moderate- to vigorous-intensity daily amount of PA for subjects from 5 to 17 years old in multiple short bouts during the day [120]. These activities include play, games, sports, active transportation, recreation, and physical education as well as planned exercise or training sessions [121]. The daily routine activities, both structured and unstructured, should be enjoyable and sustainable [122]. Moreover, the addition of resistance strength training is also recommended in order to ameliorate muscular strength and bone health in children and adolescents [120].

Despite established benefits of regular PA practice [18,123], children with obesity usually have lower levels of PA and physical fitness compared to peers [124,125] due to greater difficulties in performing different motor skills and negative feelings and self-esteem related to PA practice [126,127]. As demonstrated by Yu et al. [128], pediatric subjects with obesity spent 30% less time engaging in PA pursuits and 51% more time in sedentary behaviors during time dedicated to waking activities. Moreover, Babic et al. [129] reported that a higher physical self-esteem level in adolescents has been associated with more probability to engage in PA; conversely, a perception of low performance limits participation in PA/exercise programs and the consequent enjoyment [130,131,132]. Vandoni et al. [16] previously showed the crucial role of assessing the physical self-esteem and PA pleasure in children and adolescents before engaging in a regular exercise program. An accurate self-reported physical fitness evaluation could identify low-fitness (and possibly low-activity) children to enact surveillance and design dedicated PA interventions to increase long-time PA adherence. Moreover, across ages, several children and adolescents with obesity encountered barriers related to demographic, personal, social, and environmental factors that limit their exercise practice and PA goals. Indeed, the exercise habits of the youngsters and the amount of the exercise done by the parents influence preschool-aged children’s lifestyle [133]. Conversely, children who spend more time indoors are less active than those who spend more time outside [134]. Additionally, urban environments contribute to spending less time outdoors, and the perception of the lack of access to playgrounds, supervision, and equipment contribute to spending less time outside as well [135]. For these reasons, an active lifestyle should be promoted early both in children and in parents by intervening on family-based exercise habits that could improve children’s future fitness. Additionally, gender differences in PA level across age were found by Riddoch et al. [136], showing boys more active than girls; in particular, 21% of the children were more active at the age of 9 years old and 26% at the age of 15 years old, and gender differences in actively spent time in activities of moderate intensity were even more noticeable (20% and 36% of difference of less activity in girls). In a research by Garaulet et al. [137], with participants from 14 to 18 years old, girls with overweight had comparable levels of PA with their non-overweight counterparts, but overweight boys had a lower sport participation rate compared to their peers without overweight. In fact, sports clubs usually are too competition-oriented and hence do not include less-fit youth or particular subjects with obesity. Indeed, commonly, children and adolescents with obesity show low levels in sport participation, and the encouragement to engage in activities is crucial for the prevention and treatment of weight gain from an early age.

In summary, positive predictors of PA level include familiar context, peer, stimuli from community, an active and sportive self-identity and attitude, personal achievement [138], and perceived competence [139]. Public policies should enhance programs aimed to play outside, play at recess, and walk or bike to school. Finally, pediatricians and sport specialists should carefully evaluate barriers to the practice of exercise and implement PA and sports strategies to augment long-term PA adherence.

## 5. The Effects of Physical Exercise on NAFLD

### 5.1. Exercise Intervention in NAFLD Patients

Lifestyle intervention is considered the first line, as it has no side effects and confers multiple cardiometabolic benefits [6,107,140]. Diet- and exercise-intervention-based reduction of body weight has been shown to be effective in reducing hepatic fat deposition in children and adolescents with obesity and NAFLD [141].

Exercise is beneficial both in the prevention and in the treatment of NAFLD in adulthood, aside from dietary changes and independently from weight loss [142,143]. Indeed, it has been shown that hepatic steatosis prevalence is significantly lower in physically active men who regularly exercise (≥2–3 days per week) with respect to men with sedentary lifestyle [140]. A significant inverse association between physical activity (PA) and intrahepatic lipid content was also shown in both men and women independently of age and BMI [144]. In addition, regular exercise was significantly correlated to decreased AST, ALT, gamma-glutamyl transferase (GGT), total cholesterol (TC), triglycerides (TG), and low-density lipoproteins (LDL) in adults affected by NAFLD [143]. Although exercise is universally recommended for the treatment of NAFLD [26,50], and sedentary lifestyle is considered a risk factor for this condition during childhood [46,145], no randomized clinical trials on exercise alone versus usual care were found in children with NAFLD. Thus, for children affected by NAFLD, as for all children, WHO recommends 60 min of daily moderate- to vigorous-intensity PA and at least 3 days per week of muscle-strengthening [146]. Unfortunately, studies evaluating the effects of exercise alone as a therapy in NAFLD in children are extremely limited, and to date, no studies have investigated exercise as a treatment for children with biopsy-proven NAFLD. The most of available research evaluated the effect of exercise on obese children as the target population and not focusing only on obese children with a clear diagnosis of NAFLD [3,7].

Different clinical trials conducted on obese pediatric populations (without a proper diagnosis of NAFLD) demonstrated beneficial effects of physical exercise on liver characteristics and metabolic factors, both as preventive strategy (in children still not diagnosed with NAFLD) and as a therapeutic aid (in children affected by NAFLD) [147,148,149,150,151].

Interestingly, Gonzalez-Ruiz et al., in a meta-analysis of 14 randomized controlled trials (RCTs) in children with overweight and obesity, evaluated the effect of exercise (aerobic, resistance, and combined activities) on liver enzymes and intrahepatic triglycerides (IHTG) [152]. Exercise programs were associated with significant reductions in IHTG and GGT levels with respect to usual care. As the study was based on obese patients with obesity without a diagnosis of NAFLD per se, no specific recommendations for NAFLD pediatric patients can be driven, and the positive effects observed could be considered an efficacious preventive measure rather than a therapeutical effect for NAFLD [152]. De Piano et al. in their RCT compared the effects of aerobic training (AT) with aerobic plus resistance training (AT+RT) in adolescents with obesity and NAFLD [153]. The authors showed that 180 min of AT+RT was superior to 180 min AT on ALT and several other non-invasive NAFLD biomarkers associated with the high disease progression risk in the pediatric population [153]. In this case, PA has a therapeutical role. Lee et al. investigated the effect of AT and RT versus control on obese children with two studies: one involving boys and one girls [148,149]. Each of the study had 44 children [148,149]. The AT was structured in three sessions of 60 min per week, with treadmill, elliptical, or stationary bike. The RT consisted of three 60-minute sessions of ten whole-body exercises per week. Changes in hepatic fat fraction (HFF) for both groups were reported. However, very few children had sufficient liver fat to be considered as having NAFLD. Moreover, whether the changes reported in HFF are clinically relevant remained not fully elucidated [148,149].

Van der Heijden et al. also evaluated the effect of aerobic and resistance exercise on children [151]; specifically, in their work on AT, children with obesity (n = 15) and normal-weight children (n = 14) were provided with a 30 min aerobic intervention twice a week for 12 weeks [151]. These subjects overall showed a decrease in the mean HFF (from 9 to 6%) but no significant change in mean ALT [151]. In the study of resistance training, 12 adolescents with obesity performed a 60 min session twice a week for 12 weeks, without significant changes in HFF. Moreover, ALT levels were evaluated neither at baseline nor in response to the intervention [150].

In a recent semi-experimental study, Iraji et al. evaluated 34 obese male adolescents with clinically defined NAFLD in order to compare liver enzymes and metabolic profile changes upon selected school-based exercise (SBE) and high-intensity interval training (HIIT) [154]. The patients were divided into three groups: the HIIT group, the SBE group, and controls [154]. BMI, body fat percentage, and waist-to-hip ratio of the participants decreased after the exercise intervention. In both groups, significant reductions in the levels of IR, TG, TC, ALT, and AST were detected [154]. Moreover, the HIIT group showed a decrease in high-density lipoprotein levels that were significantly relevant with respect to the SEB group, and both the training groups showed a significant reduction in low-density lipoprotein level with respect to controls. Indeed, the authors concluded that HIIT and SBE are equally effective in improving health parameters in obese children and adolescents affected by NAFLD [154]. This is fundamental, as it highlights not only PA as a therapeutical tool for these patients but also the fact that the proper training should be tailored on patient’s abilities and choices in order to increase exercise enjoyability and improve adherence to training [154,155].

### 5.2. Exercise in NAFLD: Mechanisms of Action

The mechanisms by which exercise acts on liver fat are still greatly unknown, and pediatric studies are missing, but different works on adults showed a decrease in the hepatic fat content upon exercise even when overall weight loss was not observed [156,157,158], which is consistent with the idea that exercise has a direct effect on the liver [156,159].

Some mechanistic studies described different pathways linking increased PA to NAFLD improvement [160]. Importantly, in NAFLD, there is a complex interaction between adipose tissue, liver, and skeletal muscles, and exercise was shown to improve IR in these organs [160,161]. An improvement of IR in peripheral tissues was correlated to a lower glucose transport to the liver and a lower free fatty acid flux to the liver, with a consequent decreased liver fat content [161,162]. Moreover, PA reduces the risk of sarcopenia, which is an important risk factor for non-obese NAFLD [160,162,163]. Exercise also improves muscle function, which may further reduce systemic IR and improve hepatic inflammation [160,161] and increases cardiorespiratory fitness, which is fundamental to reduce cardiovascular risk in patients with NAFLD [161,162].

Moreover, NAFLD oxidative stress (OS), caused by reactive oxygen species (ROS) and inflammation, causes a significant damage on hepatic cells and leads to tissue injury [159]. The major sources of ROS in NAFLD are mitochondrial abnormalities, down-regulation of several antioxidant enzymes, accumulation of leukocytes, and hepatic inflammation [159]. In this context, exercise can be useful, as it was shown to suppress OS and ROS overproduction via up-regulation of several antioxidant enzymes and anti-inflammatory mediators [159]. Several experimental studies reported that exercise can decrease ROS production and pro-inflammatory cytokines, including TNF-α and IL-1β, through the antioxidant activity improvement and acting on NF-κB pathway [159,164,165,166]. Further studies are necessary to confirm these results and drive definitive conclusions about the mechanisms by which exercise can attenuate hepatic oxidative damage in NAFLD and improve hepatic status, especially in the pediatric field.

Finally, among the mechanisms by which PA can act on NAFLD, the link with GM plays a crucial role. Indeed, accumulated data clarified that dysbiosis or disruption of the GM is associated with NAFLD in both children and adolescents [167,168,169]. Despite the absence of a consistent signature of dysbiosis in NAFLD-associated gut microbiota in children, all of the pediatric studies have found decreased α-diversity (richness and evenness), an altered β-diversity, and a significant difference in the abundance of bacteria at the phylum and genus level with respect to the microbiota of control subjects [167,169,170,171,172]. Although the mechanism through which exercise can alter the gut microbiota and ameliorate NAFLD is unclear, and pediatric studies are missing, regular exercise and a balanced diet are unequivocally beneficial in the maintenance of a healthy gut microbiota [169]. In terms of exercise, Clarke et al. evaluated 40 young athletes and showed that gut microbiota diversity is greater in athletes than in non-athletic healthy persons [173]; in addition, Allen et al. demonstrated that aerobic exercise itself—independent of diet— alters gut microbiome composition in human adults by increasing fecal concentrations of short-chain fatty acids (SCFAs) [174]. Specifically, in their study, 32 lean and obese, previously sedentary, subjects participated in 6 weeks of supervised, endurance-based exercise training (three trainings per week) that progressed from low to vigorous intensity [174]. Subsequently, participants were allowed to return to sedentary lifestyle activity for a 6-week period. Fecal samples were collected before and after the 6-week period of regular exercise and after the sedentary washout period. The fecal samples were analyzed, and exercise resulted in increased fecal concentrations of SCFAs in lean but not obese participants [174]. The exercise-induced change in metabolic output of the microbiota was consistent with the parallel shift in bacterial genes and taxa capable of SCFAs production. Interestingly, exercise-induced microbiota changes were largely reversed once exercise training stopped [174]. Quiroga et al. demonstrated that 12 weeks of exercise training favorably altered the deleterious obesity-related microbiota profile and reduced microbial inflammatory signaling in children with obesity [175]. The authors enrolled 39 obese children that were randomly assigned to the control or training (combined strength and endurance exercise) group [175]. The exercise group not only had reduced blood glucose and GOT levels with respect to controls but also significantly decreased abundance of the obesity-related Proteobacteria phylum and Gammaproteobacteria class and increased levels of some beneficial bacterial genera, such as Blautia, Dialister, and SCFAs-producing Roseburia [175].

These findings suggest that exercise can positively modify the deleterious microbiota profile present in obese subjects affected by NAFLD. Nevertheless, additional research is needed to understand how exercise determines these changes and all their related effects both in terms of NAFLD prevention and therapy.

**Figure 3:**
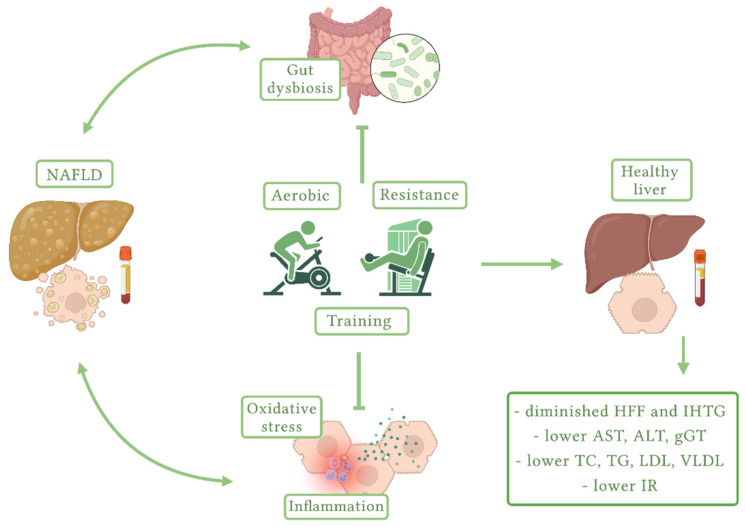
Benefits of exercise on NAFLD (created with BioRender.com, accessed on 1 July 2022). NAFLD, non-alcoholic fatty liver disease; HFF, hepatic fat fraction; IHTG, intrahepatic triglycerides; AST, aspartate aminotransferase; ALT, alanine aminotransferase; gGT, gamma-glutamyl transferase; TC, total cholesterol; TG, triglycerides; LDL, low-density lipoprotein; VLDL, very-low-density lipoprotein; IR, insulin resistance.

Benefits of exercise on NAFLD are schematized in Figure 3.

## 6. Conclusions

As sedentary behavior is a risk factor for NAFLD development independent from weight gain, tailored exercise has been highlighted as effective strategy to prevent and treat NAFLD. Several studies on obese children and adults confirm the positive role of PA in the treatment of NAFLD, but to date, there are no pediatric RCTs on exercise versus usual care. Moreover, literature concerning the prognostic value of exercise in the treatment of pediatric NAFLD is missing. Among the mechanisms involved in the effects of PA on NAFLD, the main players seem to be IR and related inflammation, oxidative stress, and gut dysbiosis, but further evaluations are necessary to deeply understand whether these factors are correlated and how they synergistically act. Thus, deeper research on this theme is needed, and it would be extremely interesting.

## Data Availability

Not applicable.
